# Supplementary material for: LEMMIv2: benchmarking framework for metagenomic and 16S amplicon profilers with a catalogue of evaluated tools
Source: Genome Biol. 2026 Apr 28;27:198. doi: 10.1186/s13059-026-04089-9 (PMC13270737; doi:10.1186/s13059-026-04089-9)
Supplement: Supplementary file 1 — Additional file 1. Supplementary Material to Seppey et al. 2026 - LEMMIv2: benchmarking framework for metagenomic and 16S amplicon profilers with a catalogue of evaluated tools. Description: All supplementary figures and tables. [file 13059_2026_4089_MOESM1_ESM.docx]

Supplementary material to Seppey et al. 2026 - LEMMIv2: benchmarking framework for metagenomic and 16S amplicon profilers with a catalogue of evaluated tools.

Figures: pages 1- 9

Tables: pages 10 - 12


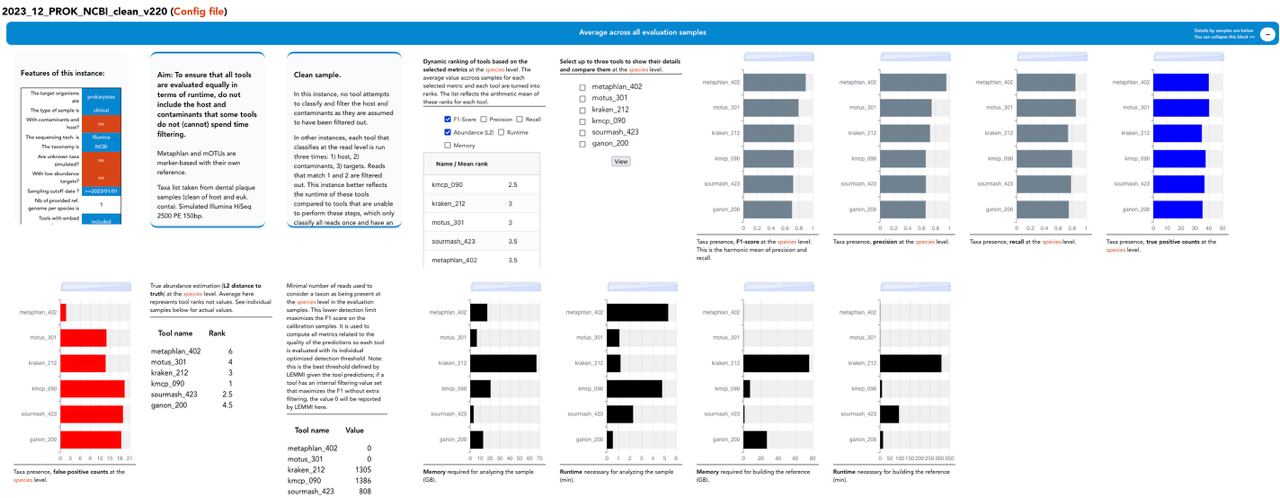


**Fig. S1.** Detail page of a LEMMIv2 instance, showing different widgets reporting average values for all the samples and metrics that are common to all samples such as resources for building the reference.


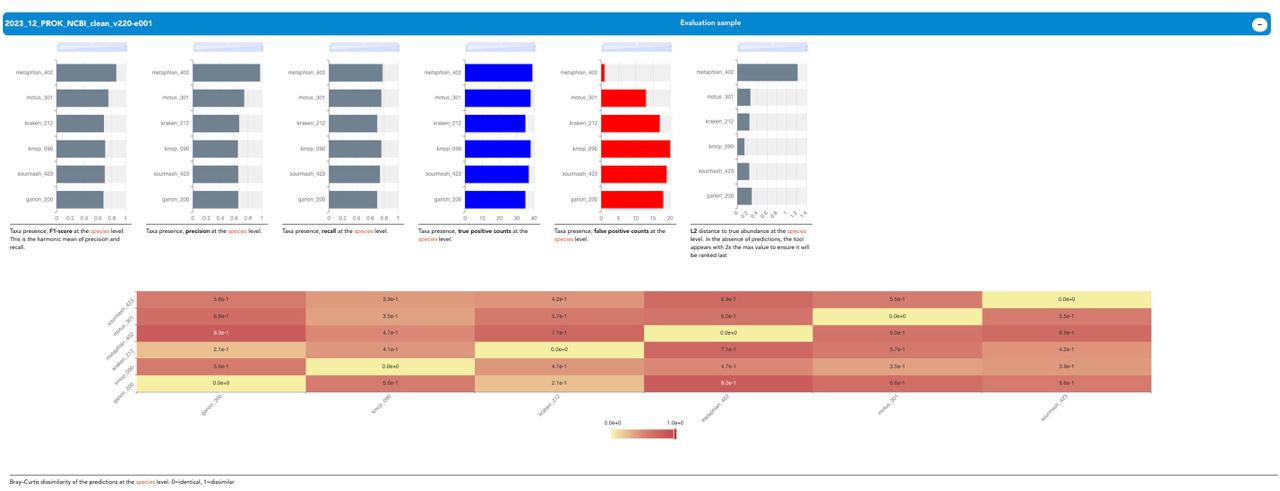


**Fig. S2.** Detail page of a LEMMIv2 instance, showing different widgets reporting values for a specific evaluation sample, including a heatmap on how dissimilar the predictions of each tool are for that sample.


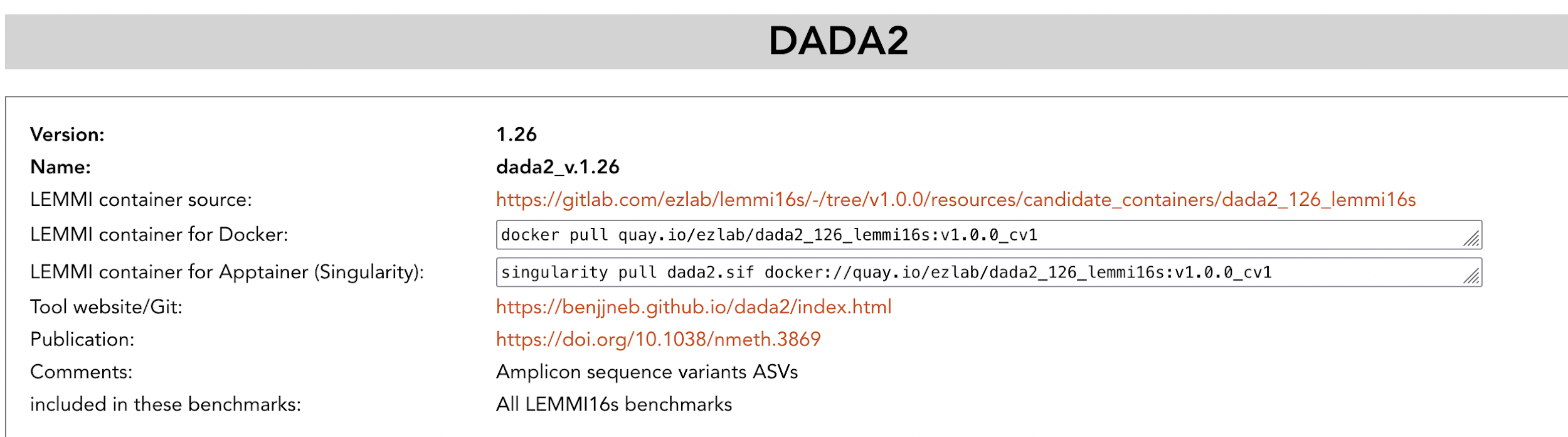


**Fig. S3.** Extract from the LEMMI16S catalogue. Each tool has a section where different versions can be presented, with a link to the source of the container, the built container available in a repository, and links to a website and publication for the method where applicable.


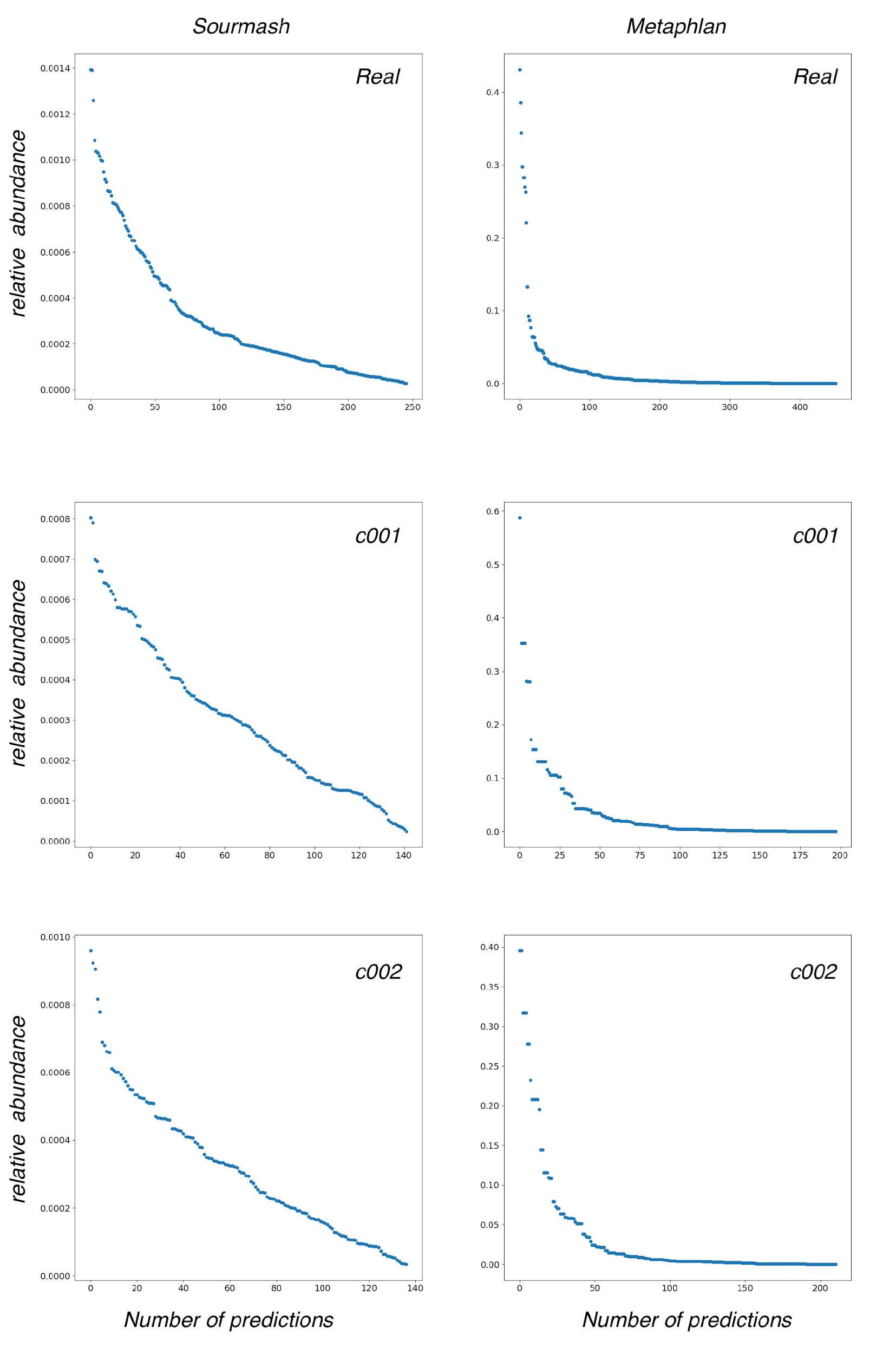


**Fig. S4.** Relative abundances of taxa predicted by Sourmash and Metaphlan on LEMMIv2 samples 2023_05_PROK_NCBI-c001, c002 and the corresponding real sample. Includes predictions at all taxonomic ranks.

**Fig. S5.** Performance of methods for the identification of organisms at species level for the instance 2023_12_PROK_NCBI_v220. Methods marked with _all used up to five genomes for each species as a reference. The others used only one genome as reference.

**Fig. S6.** Performance of methods for the identification of organisms at genus level for the instance 2023_12_PROK_NCBI_v220. Methods marked with _all used up to five genomes for each species as a reference. The others used only one genome as reference.

**Fig. S7.** A) Average runtime (minutes) to perform the analysis of a sample from the 2023_12_PROK_NCBI_clean_v220 instance. B) Average peak memory (GB) to perform the analysis of a sample from the 2023_12_PROK_NCBI_clean_v220 instance. C) F1 score of methods for the identification of organisms at species level for the instance 2023_11_PROK_GTDB_v220. D) F1 score of methods for the identification of organisms at species level for the instance 2022_03_PROK_NCBI_2. E) F1 score of methods for the identification of organisms at species level for the instance 2022_01_EUK_NCBI. F) F1 score of methods for the identification of organisms at species level for the instance 2021_9_VIR_NCBI. G) F1 score of methods for the identification of organisms at species level for the instance 2022_04_PROK_NCBI_LR2. H) Recall of methods for the identification of organisms at species level for the instance 2025_09_PROK_NCBI_PACBIO_v220.


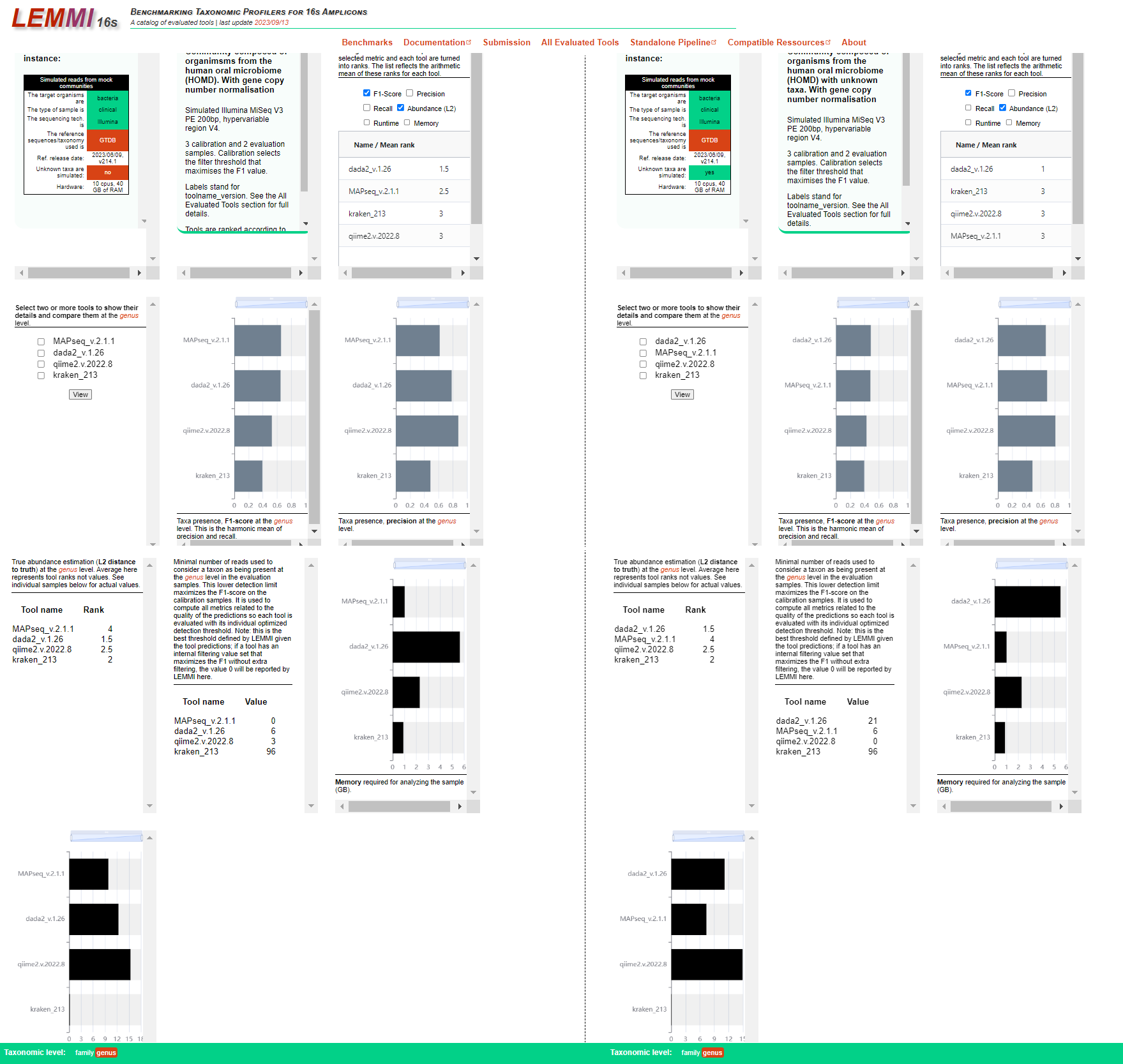


**Fig. S8.** Results taken from LEMMI16S instance representing Human Oral Microbiome samples with unknown organisms.

**Table S1:** Characteristics of simulated samples compared to the real samples on which they are based.

|  | **Reads**  **length** | **Number of paired-end reads** | **Number of 31-mers** | **%GC** |
| --- | --- | --- | --- | --- |
| **LEMMIv2** |  |  | | |
| Plaque from a healthy patient | 151 | 21,153,159 | 2,059,607,399 | 45 |
| Simulation  2023_05_PROK_NCBI-c001 | 150 | 20,015,528 | 2,206,174,136 | 41 |
| Simulation  2023_05_PROK_NCBI-c002 | 150 | 20,055,700 | 2,224,328,630 | 41 |
| **LEMMI16S** |  |  |  |  |
| Human pathogens samples | 300 | 654,196 | 354,293,576 | 53.7 |
| Simulation  Human pathogens samples_gcn-c001 | 250 | 867,086 | 380,472,649 | 52.4 |
| Simulation Human pathogens samples_gcn-c002 | 250 | 892,299 | 392,439,207 | 53.6 |

**Table S2:** Status of the tools in terms of the ability of the container to filter out off-target reads prior to final classification. In some cases, the tool does not classify at the read level, making such pre-filtering impossible, and in other cases, the process was considered too heavy in terms of runtime and memory.

| **Tool name** | **Filter host** | **Filter contaminants** |
| --- | --- | --- |
| ccmetagen_140 | Yes | No |
| centrifuge_104 | Yes | Yes |
| centrifuge_100 | Yes | Yes |
| deepmicrobes_g | No | No |
| ganon_200, _160, _100 | Yes | Yes |
| kmcp_090, _081 | Yes | Yes |
| kraken_212 | Yes | Yes |
| metabuli_101 | Yes | Yes |
| metacache_220 | Yes | Yes |
| metamaps_01 | Yes | Yes |
| metaphlan_402, _3013 | No | No |
| mmseqs_12113 | Yes | Yes |
| motus_301 | No | No |
| singlem_0190_r207, _r226 | No | No |
| sourmash_423 | Yes | Yes |
| virmet_100 | Yes | Yes |

**Table S3:** Estimation of true abundance (L2 distance to truth) at species level. Average ranks in minimising error across all evaluation samples from instance 2023_12_PROK_NCBI_v220.

| **Tool name** | **Value** |
| --- | --- |
| metaphlan_402 | 1.5 |
| motus_301 | 5.25 |
| kraken_212 | 6.75 |
| kraken_212_all | 4.75 |
| ganon_200 | 8.5 |
| sourmash_423_all | 12 |
| ganon_200_all | 7 |
| kmcp_090 | 2.5 |
| sourmash_423 | 13 |
| singlem_0190_r207 | 3 |
| centrifuge_104 | 6.5 |
| metabuli_102 | 9.75 |
| centrifuger_100 | 11 |
